# Incomplete Abortion in Twin Pregnancy

**Published:** 1887-07

**Authors:** 


Incomplete Abortion in Twin Pregnancy.—Dr. Stanly P. Warren, of Portland, Maine, reports a case of twin pregnancy in which one child was lost at three months, but its placenta remained to delivery of the other twin at term. The twin at term was a healthy, robust child, weighing three pounds. It is rare, if not unique.

